# Elevated Ras related GTP binding B (RRAGB) expression predicts poor overall survival and constructs a prognostic nomogram for colon adenocarcinoma

**DOI:** 10.1080/21655979.2021.1956402

**Published:** 2021-07-29

**Authors:** Jianjia Xiao, Qingqing Liu, Weijie Wu, Ying Yuan, Jie Zhou, Jieyu Shi, Shaorong Zhou

**Affiliations:** a Department of General Surgery, Taizhou Hospital of Traditional Chinese Medicine, Taizhou, Jiangsu Province, China; b Department of Gastroenterology, Affiliated Hospital NO.2 Of Nantong University, Nantong, Jiangsu Province, China; c Department of Orthopedics, The Sixth People’s Hospital of Nantong, Medical College of Nantong University, Nantong, Jiangsu Province, China; d Department of Geriatrics, Taizhou Second People’s Hospital, Taizhou, Jiangsu Province, China; e Department of Neurology, Taizhou Hospital of Traditional Chinese Medicine, Taizhou, Jiangsu Province, China

**Keywords:** Colon adenocarcinoma, RRAGB, nomogram, survival, TCGA

## Abstract

Currently, no articles have explored the roles of RRAGB gene in the occurrence and development of cancer. By means of The Cancer Genome Atlas (TCGA) data mining, we found that this gene might be a novel prognostic predictor for colon adenocarcinoma (COAD). Hence, this article was carried out to explore its roles in COAD and associations with immunity. RRAGB single-gene expression matrix and corresponding clinical information were extracted from TCGA database. Univariate/multivariate cox regression analyses and gene set enrichment analysis (GSEA) were utilized to identify independent prognostic factors and RRAGB related pathways, respectively. Relationships between RRAGB and immunity were also analyzed. Boxplot and K-M survival analysis indicated that RRAGB was not only differently expressed in COAD (*P* < 0.05), but also significantly associated with overall survival (OS; *P* < 0.05). Univariate and multivariate Cox hazard regression analyses indicated that RRAGB could serve as an independent prognostic factor for COAD (both *P* < 0.05). GSEA identified five signaling pathways significantly enriched in the high-RRAGB expression phenotype. Moreover, a RRAGB-based nomogram was successfully constructed and displayed a satisfactory performance. In addition, RRAGB expression was found to be significantly associated with microsatellite instability (MSI), tumor mutational burden (TMB) and immunity. Our results revealed that RRAGB could be a prognostic biomarker for COAD in terms of OS and markedly related to MSI, TMB, and immunity. We also constructed an RRAGB-based nomogram with a satisfactory performance. Further researches should be carried out to validate our findings.

## GRAPHICAL ABSTRACT

**Figure:**
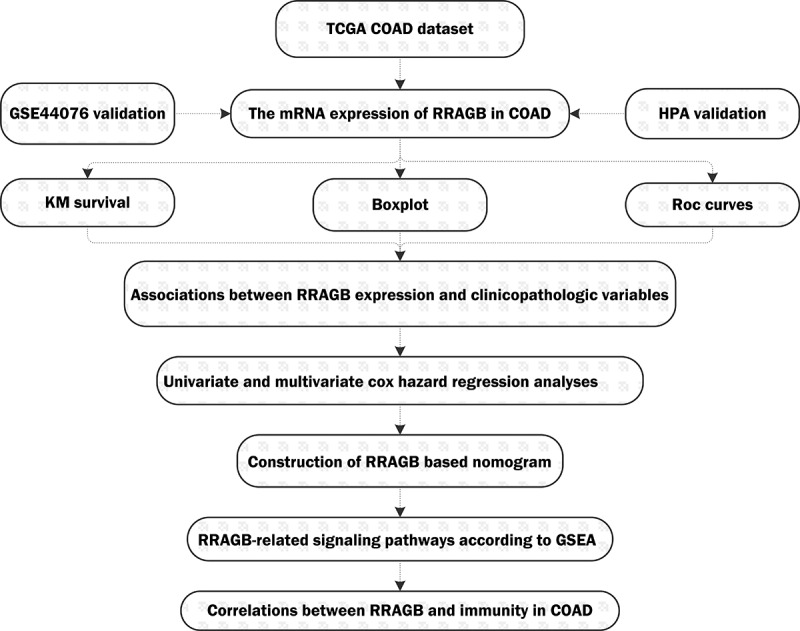

## Introduction

Colorectal cancer (CRC), as one of the most common tumor diagnosed in the digestive system, accounts for 8% with approximately 149,500 newly diagnosed cases and accounts for 9% with approximately 52,980 newly estimated death in the United States, 2021 [1]. Colon adenocarcinoma (COAD), as the most common histological subtype of colon cancer, mainly occurs in the intestinal mucosa and spreads to adjacent organs [2]. Although the 5-year survival rate for early-stage colon cancer patients undergoing radical resection is more than 90%, most patients are diagnosed with advanced cases or metastasis, leading to the drop of 5-year survival rates to 10% [3]. Currently, surgery, radiotherapy, chemotherapy, targeted therapies are available for the clinical treatment of COAD and considerable advancements have been achieved in these therapeutic strategies [4]. However, patients’ prognosis is still poor and far from satisfactory due to the late diagnosis, rapid development and high frequency of metastasis [5,6]. Hence, there is an urgent need to explore the molecular mechanisms of COAD and to identify novel biomarkers for survival evaluation and targeted treatment [7].

Ras-related GTP binding B (RRAGB), also known as RAGB or bA465E19.1, belongs to the large family of Ras-homologous GTPases and encodes proteins of cellular switches operated by GTP-exchange factors and factors stimulating their intrinsic GTPase activity [8,9]. Thanks to the great improvement of high-throughput sequencing technology and an increasing establishment of public database networks, more and more transcriptome and clinical data could be explored by means of bioinformatics analysis. Based on these, a growing number of prognostic biomarkers or signatures had been established in different cancers [10–12]. As for RRAGB, Shi et al. successfully constructed a signature based on CHMP4C, FOXO1, RRAGB to effectively predict the cervical cancer patients’ prognosis [13] and Xie et al. also constructed a six-gene model including RRAGB predict the non-small-cell lung cancer patients’ overall survival (OS) [14]. Currently, little was still known about the roles of RRAGB gene in the occurrence and development of COAD. In this article, we aimed to explore the comprehensive roles of RRAGB in COAD and to underline its associations with immunity, hoping to provide a novel candidate gene to improve the prognosis and survival rates of COAD.

## Materials and methods

### Single gene matrix mining from the TCGA database

RNA-sequencing FPKM data and corresponding clinical information of 39 normal and 398 COAD tumor tissues were extracted from the official website of The Cancer Genome Atlas (TCGA; https://tcga-data.nci.nih.gov/tcga/). R version 3.5.1 software (https://www.r-project.org/) was utilized to standardize RRAGB RNA-Sequencing data [15] and we further did an overlap with RRAGB mRNA to get single-gene expression matrix and clinical information for each sample ID. OS was the primary outcome of this study and we further analyzed the associations between RRAGB’s expression and clinical data. Besides, we also used ‘limma’ package (http://www.bioconductor.org/packages/release/bioc/html/limma.html) to calculate differently expressed genes (DEGs), with the cutoff criteria of adjusted P-value (FDR) <0.05 and |log2 FC (fold change)| ≥1 [16].

### Gene set enrichment analysis (GSEA)

As a reference, the ‘c2.cp.kegg.v6.2.symbols.gmt’ gene set was obtained from the Molecular Signatures Database (MSigDB) (http://software.broadinstitute.org/gsea/msigdb) [17]. We performed GSEA to reveal significant survival differences between the high-RRAGB and low-RRAGB expression groups, with at least 1000 times permutation tests for each analysis [18]. Through this way we could discover significant critical biological pathways, with the threshold of the normalized enrichment score (NES)>1.5 and normal p values <0.05.

### Protein–protein interaction (PPI) network and the Human Protein Atlas (HPA) database

The PPI network analysis was carried out with the help of the online STRING (https://string-db.org/) website for the sake of exploring the potential relationships between RRAGB and other genes [19]. We explored the online HPA (http://www.proteinatlas.org/) database to validate the RRAGB protein expression of COAD through immunohistochemical staining by antibody HPA003734 [20].

### Sangerbox website tools

As a free data analysis platform, the Sangerbox website tools (http://www.sangerbox.com/tool) [21,22] was utilized by us to explore the correlations between the RRAGB expression and microsatellite instability (MSI), tumor mutational burden (TMB), tumor neoantigen burden (TNB), tumor microenvironment, tumor immune infiltration, immune checkpoint molecules, immune cells pathway, mismatch repair genes by the Spearman’s or the pearson’s method [23–25]. All indicators were visualized by R-packages (https://www.r-project.org/) and *P* < 0.001 was set as the cutoff threshold.

### Statistical analysis

All statistical analyses were conducted by using R version 3.5.1 software (https://www.r-project.org/). The ‘limma’ package (http://www.bioconductor.org/packages/release/bioc/html/limma.html) was used to compare the RRAGB expression differences between the normal and cancer tissues by Student’s t test [26]. The Wilcoxon signed-rank test [27] and logistic regression [28] were also performed by us to estimate the associations between the RRAGB expression and clinicopathological variables. Based on the median expression of RRAGB, COAD patients were divided into high-risk groups and low-risk groups. Survival analysis was conducted by Kaplan–Meier (K–M) method with the log-rank test [29]. The receiver operating characteristic (ROC) curves associated with the area under the curve (AUC) values were calculated by the R ‘survivalROC’ package (https://cran.r-project.org/web/packages/survivalROC/index.html). Univariate and multivariate cox hazard regression analyses were applied to identify independent prognostic factors. Furthermore, nomogram was also visualized by the R ‘rms’ package (https://cran.r-project.org/web/packages/rms/index.html) to predict the 1-, 3-, and 5-year survival probabilities and individual predictors. In addition, all P values were adopted by a two-sided test and P < 0.05 was regarded as statistical significance.

## Results

By means of TCGA data mining, we aimed to explore the comprehensive roles of RRAGB in COAD and to underline its associations with immunity, hoping to provide a novel candidate gene to improve the prognosis and survival rates of COAD. It was the first time for us to comprehensively explore the expression of RRAGB and its impact on COAD. Our results indicated that RRAGB could serve as an independent prognostic factor for predicting the prognosis of COAD patients and it was significantly associated with MSI, TMB, immunity. GSEA identified five RRAGB‑related signaling pathways and RRAGB-based nomogram was also constructed to guide the prognosis of COAD patients. Taken together, RRAGB might be a novel prognostic predictor for COAD.

### RRAGB expression levels in COAD from TCGA and HPA database

**Figure 1.**
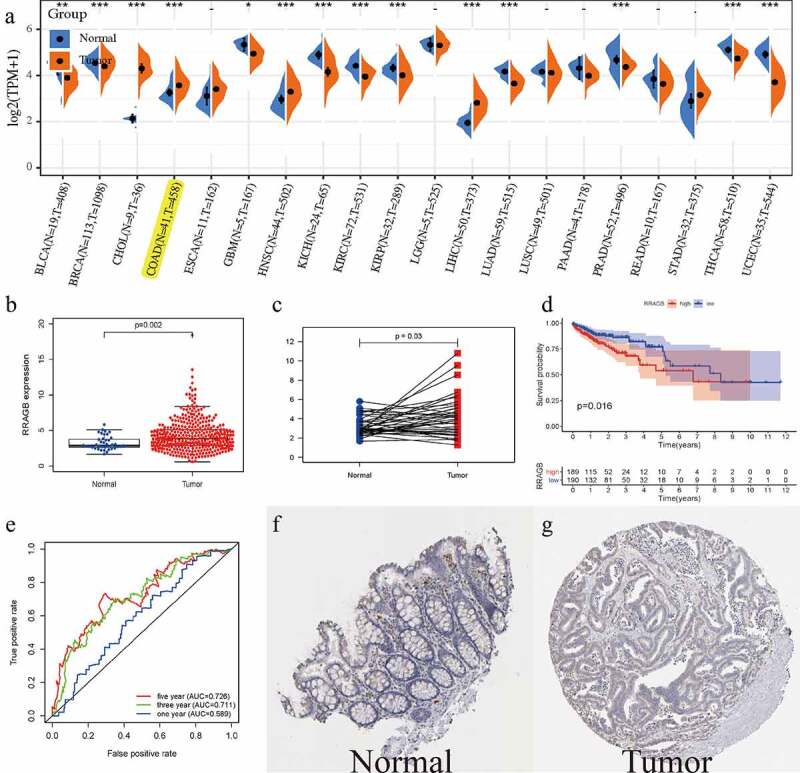
The expression levels of RRAGB in COAD tissues; (a) The mRNA expression levels of RRAGB in pan-cancers from the TCGA database; (b) The RRAGB mRNA expression by boxplot in COAD from TCGA (N = 39; T = 398); (c) The RRAGB mRNA expression by pairwise boxplot in COAD from TCGA (N = 39; T = 39); (d) K–M survival analysis of RRAGB in COAD from TCGA; (e) ROC curves and 1-, 3-, and 5-year AUCs of RRAGB in COAD from TCGA; (f-g) The protein expression levels of RRAGB by immunohistochemical staining from the HPA database; *P < 0.05; **P < 0.01; ***P < 0.001

The RRAGB mRNA expression levels in different cancer types are detailed in Figure 1(a) and we could detect that RRAGB was differently expressed in 14 cancer types including COAD (all *P* < 0.05). We then selected COAD for further analysis. Boxplot (N = 39; T = 398) and pairwise boxplot (N = 39; T = 39) confirmed that RRAGB mRNA was up-regulated in COAD tumor tissues, compared with normal tissues (both *P* < 0.05; Figure 1(b,c)). K-M survival analysis indicated that the low-RRAGB group had a much longer OS than the high-RRAGB group, based on its median expression in COAD (*P* = 0.016; Figure 1(d)). Moreover, ROC curves of RRAGB associated with 1-, 3-, and 5-year AUCs were 0.589, 0.711, 0.726, respectively (Figure 1(e)). As displayed in figure 1(f,g), immunohistochemical staining of the HPA database indicated that RRAGB was not detected in normal colon tissues and its expression was medium in COAD tumor tissues. Moreover, we also employed GSE44076 dataset to validate the expression of RRAGB in normal and tumor COAD cells (*P* = 1.92e-07; N = 148; T = 98; **Supplement Figure S1**).

### Associations between RRAGB expression and clinicopathologic variables

**Figure 2.**
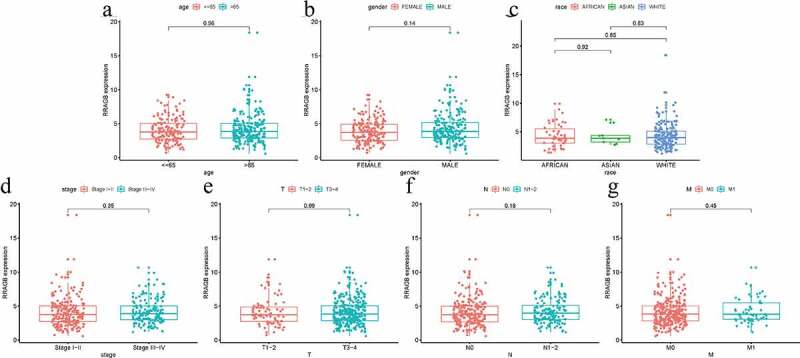
Associations between RRAGB expression and clinicopathologic variables; (a) Associations between RRAGB mRNA expression and age; (b) Associations between RRAGB mRNA expression and gender; (c) Associations between RRAGB mRNA expression and race; (d) Associations between RRAGB mRNA expression and stage; (e) Associations between RRAGB mRNA expression and T stage; (f) Associations between RRAGB mRNA expression and N stage; (g) Associations between RRAGB mRNA expression and M stage

The Wilcoxon signed-rank test and logistic regression were performed by us to estimate the associations between the RRAGB expression and seven clinicopathological variables including age, gender, race, stage, T, N, M. However, no significant associations were observed (all *P* > 0.05, Figure 2).

### Univariate and multivariate cox hazard regression analyses

**Figure 3.**
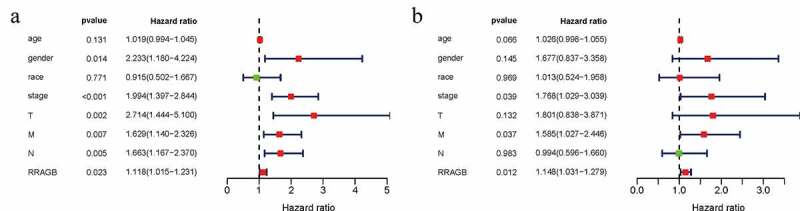
Univariate and multivariate cox hazard regression analyses; (a) Univariate Cox hazard regression analysis of eight clinicopathological variables (RRAGB, age, gender, race, stage, T, N, M) in COAD from TCGA dataset; (b) Multivariate Cox hazard regression analysis of eight clinicopathological variables (RRAGB, age, gender, race, stage, T, N, M) in COAD from TCGA dataset

Univariate and multivariate Cox hazard regression analyses were applied by us to identify independent prognostic factors from RRAGB, age, gender, race, stage, T, N and M. Univariate Cox hazard regression analysis showed that gender, stage, T, N, M and RRAGB were all significantly related to OS (all *P* < 0.05; Figure 3(a) and Table 1). Multivariate Cox hazard regression analysis presented that stage, M and RRAGB were markedly linked to the OS (all *P* < 0.05; Figure 3(b) and Table 1). In other words, stage, M and RRAGB were all independent prognostic factors for COAD.

**Table 1.** Univariate and multivariate cox hazard regression analyses of COAD in TCGA

Characteristics	Univariate analysis				Multivariate analysis			
	HR	HR.95 L	HR.95 H	pvalue	HR	HR.95 L	HR.95 H	pvalue
**Age**	1.019482	0.994269	1.045334	0.131014	1.026415	0.998231	1.055396	0.06646
**Gender**	2.23311	1.180471	4.224399	**0.01351**	1.676718	0.837335	3.357536	0.144605
**Race**	0.914905	0.502185	1.666818	0.771367	1.013196	0.524169	1.958468	0.9689
**Stage**	1.993568	1.397495	2.843882	**0.00014**	1.768459	1.029165	3.038821	**0.03901**
**T**	2.713683	1.444052	5.099591	**0.00192**	1.800819	0.837723	3.871144	0.131941
**M**	1.628803	1.14046	2.326253	**0.0073**	1.585269	1.027464	2.445903	**0.0373**
**N**	1.663132	1.167274	2.369631	**0.00486**	0.994441	0.59583	1.659724	0.982982
**RRAGB**	1.118193	1.015388	1.231407	**0.02319**	1.148259	1.030556	1.279405	**0.01223**

### Construction of RRAGB based nomogram

**Figure 4.**
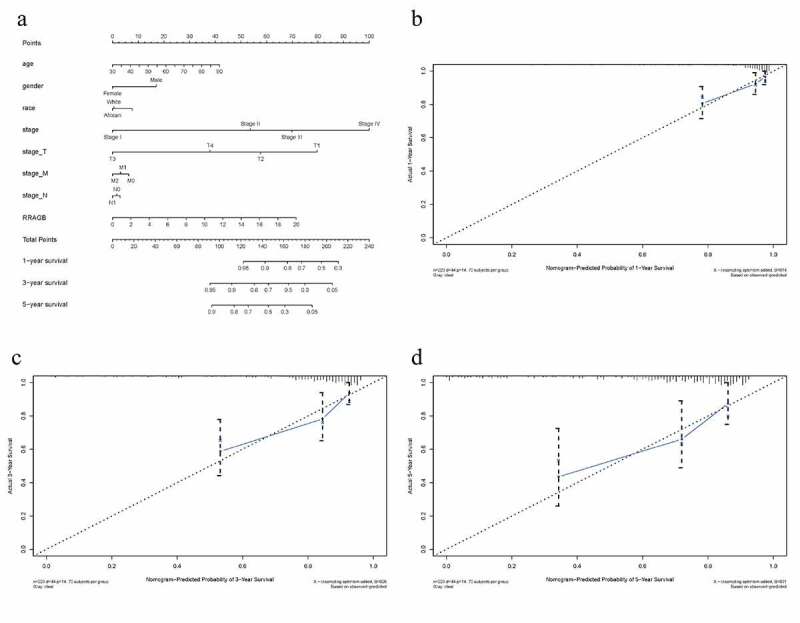
RRAGB based nomogram and evaluation; (a) Nomogram based on eight clinicopathological variables (RRAGB, age, gender, race, stage, T, N, M) in COAD from TCGA dataset; (b) 1-year calibration curve; (c) 3-year calibration curve; (d) 5-year calibration curve

A nomogram was constructed by the R ‘rms’ package to intuitively visualize the associations between eight clinicopathological variables (RRAGB, age, gender, race, stage, T, N and M) and 1-, 3-, 5-year survival probabilities of OS (Figure 4(a)). As summarized in Table 2, C-index and 1-, 3-, 5-year AUCs of RRAGB-based nomogram were 0.872, 0.748, 0.799 and 0.791, showing a moderate prediction accuracy. As displayed in Figure 4(b–d), 1-, 3-, and 5-year calibration curves indicated the consistency of our results and the predictive values, indicating satisfactory performance for this RRAGB-based nomogram.

**Table 2.** C-index and 1-, 3-, 5-year AUCs of RRAGB-based nomogram

	1-year	3-year	5-year	C-index
**AUC**	0.748	0.799	0.791	0.872

### RRAGB‑related signaling pathways according to GSEA

**Figure 5.**
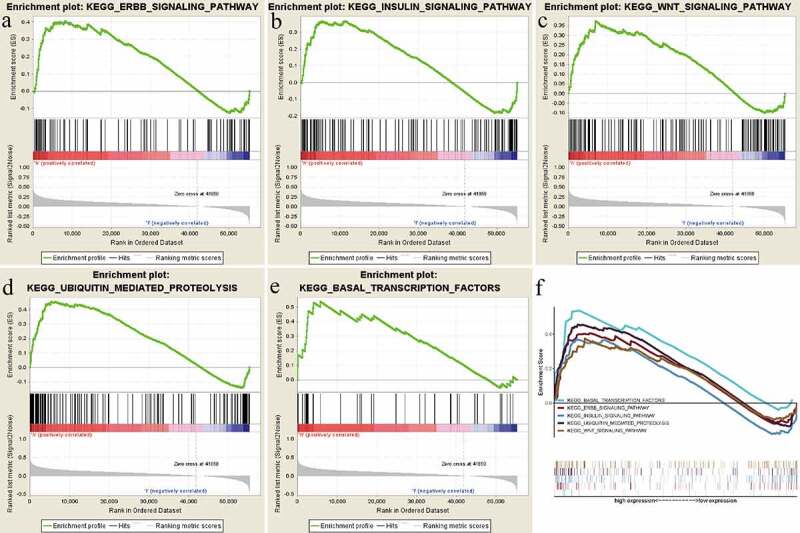
Gene set enrichment analysis (GSEA) results based on the RRAGB mRNA expression in COAD from TCGA dataset; (a) Significantly enriched pathway of Erbb signaling pathway; (b) Significantly enriched pathway of Insulin signaling pathway; (c) Significantly enriched pathway of Wnt signaling pathway; (d) Significantly enriched pathway of Ubiquitin mediated proteolysis; (e) Significantly enriched pathway of Basal transcription factors; (f) All of these five significantly enriched signaling pathways

GSEA was by us performed to reveal significant survival differences between the high-RRAGB and low-RRAGB expression groups to discover significant critical biological pathways, based on the the ‘c2.cp.kegg.v6.2.symbols.gmt’ gene set from the MSigDB. Our results indicated that the high-RRAGB expression phenotype was significantly associated with Basal transcription factors, Ubiquitin mediated proteolysis, Insulin, Wnt, Erbb signaling pathways, with the threshold of NES >1.5 and normal p values <0.05 (Figure 5 and Table 3).

**Table 3.** Gene set enrichment analysis results

MSigDB collection	Gene set name	NES	NOM p-val	FDR q-val
c2.cp.kegg.v7.1.symbols.gmt	KEGG_ERBB_SIGNALING_PATHWAY	1.524	0.040	0.603
	KEGG_INSULIN_SIGNALING_PATHWAY	1.516	0.029	0.501
	KEGG_BASAL_TRANSCRIPTION_FACTORS	1.675	0.029	1.000
	KEGG_UBIQUITIN_MEDIATED_PROTEOLYSIS	1.759	0.012	1.000
	KEGG_WNT_SIGNALING_PATHWAY	1.535	0.037	0.764

### Associations between RRAGB and PPI network, MSI, TNB, TMB in COAD

**Figure 6.**
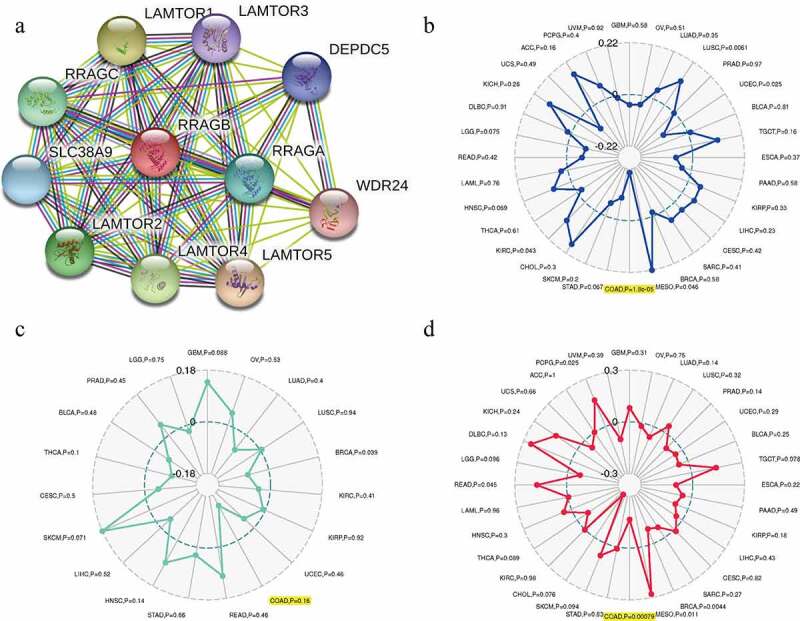
Associations between RRAGB and PPI network, MSI, TNB, TMB in COAD; (a) PPI network based on RRAGB expression; (b) Associations between RRAGB expression and MSI in COAD; (c) Associations between RRAGB expression and TNB in COAD; (d) Associations between RRAGB expression and TMB in COAD

A PPI network analysis was carried out with the help of the online STRING (https://string-db.org/) website to explore the potential relationships between RRAGB and other genes in COAD (Figure 6(a)). By means of the pearson’s method, we calculated the correlations between the RRAGB expression and MSI, TNB, TMB, with the help of the Sangerbox website tools (http://www.sangerbox.com/tool). Radar maps indicated that the RRAGB expression was dramatically linked to MSI (*P* = 1.8e-05) and TMB (*P* = 0.00079) in COAD, whereas it was not associated with TNB (*P* = 0.16).

### Associations between RRAGB and tumor immune infiltration, SCNA, tumor microenvironment in COAD

**Figure 7.**
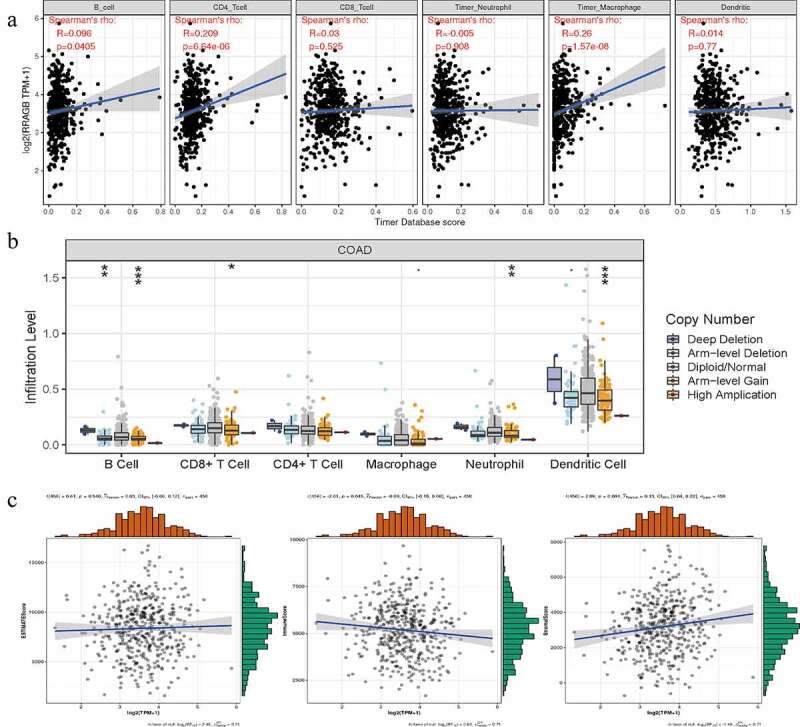
Associations between RRAGB expression and tumor immune infiltration, SCNA, tumor microenvironment in COAD; (a) Associations between RRAGB expression and tumor immune infiltration in COAD from TCGA dataset; (b) Associations between RRAGB expression and SCNA in COAD from TCGA dataset; (c) Associations between RRAGB expression and tumor microenvironment in COAD from TCGA dataset; *P < 0.05; **P < 0.05; ***P < 0.001

With the help of the Sangerbox website tools, we calculated the correlations between the RRAGB expression and tumor immune infiltration by means of the Spearman’s method. The RRAGB mRNA expression was markedly related to B cells, CD4 + T cells and Macrophage cells infiltration (all *P* < 0.05, Figure 7(a)). SCNA module provided the correlations between tumor immune infiltration levels among COAD and different somatic copy number alterations for RRAGB by the Wilcoxon rank-sum test (Figure 7(b)). With the pearson’s method, the RRAGB mRNA expression was significantly associated with ImmuneScore and StromalScore (both *P* < 0.05), whereas it was not linked to ESTIMATEScore (*P* = 0.540, Figure 7(c)).

### Correlations between RRAGB and immune checkpoint molecules, immune cells pathway, mismatch repair genes in COAD

**Figure 8.**
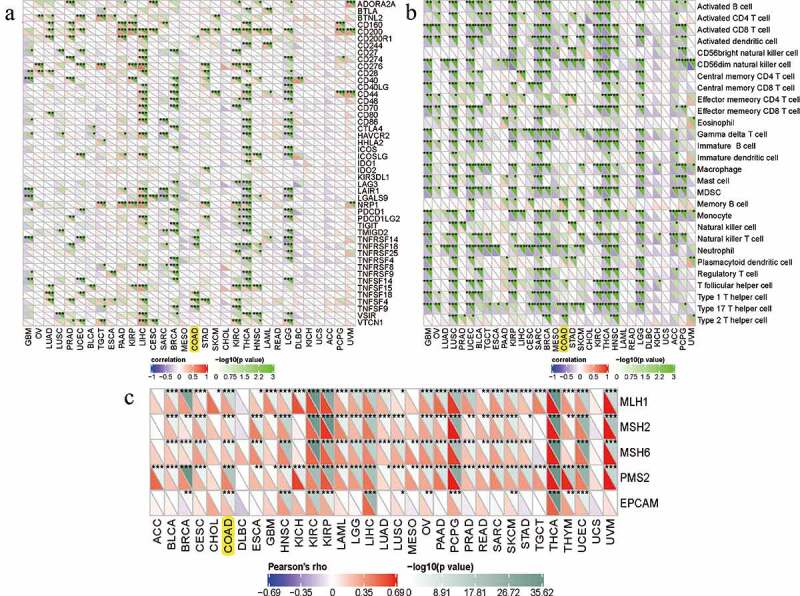
Correlations between RRAGB expression and immune checkpoint molecules, immune cells pathway, mismatch repair genes in COAD; (a) Correlations between RRAGB expression and immune checkpoint molecules in COAD from TCGA dataset; (b) Correlations between RRAGB expression and immune cells pathway in COAD from TCGA dataset; (c) Correlations between RRAGB expression and mismatch repair genes in COAD from TCGA dataset; *P < 0.05; **P < 0.05; ***P < 0.001

By means of the pearson’s method, we calculated the correlations between the RRAGB expression and immune checkpoint molecules, immune cells pathway, mismatch repair genes, with the help of the Sangerbox website tools. As for immune checkpoint molecules, the RRAGB mRNA expression was significantly associated with TNFSF4, TNFSF9, TNFSF18, TMIGD2, TNFRSF14, TNFRSF18 in COAD (all *P* < 0.05, Figure 8(a)). In terms of immune cells pathways, the RRAGB mRNA expression was markedly related to Type 17 T helper cell, Type 2 T helper cell, Neutrophil, Monocyte, Memory B cell, CD56dim natural killer cell, Activated dendritic cell and so on in COAD (all *P* < 0.05, Figure 8(b)). Co-expression analysis of RRAGB and mismatch repair genes indicated that RRAGB was dramatically linked to MLH1, MSH2, MSH6, PMS2, EPCAM in COAD (all *P* < 0.001, Figure 8(c)).

## Discussion

Currently, no studies have reported the definite roles of RRAGB in human cancers and it was the first time for us to comprehensively explore the expression of RRAGB and its impact on COAD. In this article, our results indicated that RRAGB was not only differently expressed in COAD, but also significantly associated with OS. Further univariate and multivariate Cox hazard regression analyses indicated that stage, M and RRAGB could all serve as independent prognostic factors for COAD. As reported by previous researches, Bunte et al. found that RRAGB was more prevalent in periodontitis patients than healthy controls and might be associated with periodontitis clinical manifestations [30]. Shi et al. successfully constructed a signature based on CHMP4C, FOXO1, RRAGB, and effectively predicted the cervical cancer patients’ prognosis [13]. Xie et al. also constructed a six-gene model including RRAGB and it could function as an independent prognostic factor to predict the non-small-cell lung cancer patients’ OS [14].

In this article, our results indicated that the high-RRAGB expression phenotype was significantly associated with Basal transcription factors, Ubiquitin-mediated proteolysis, Insulin, Wnt, Erbb signaling pathways. As a useful tool, GSEA had been applied by various studies to reveal significant survival differences between the high- and low-gene expression groups to discover significant critical biological pathways [31]. Shuai et al found that eight signaling pathways (apoptosis, cell cycle, ErbB, MAPK, mTOR, Notch, p53 and TGF-β pathways) were most significantly enriched in the high-CDCA8 phenotype, according to the GSEA [11]. Liu et al. reveal that ZNF132 could participate in multiple biological pathways containing the regulation of glycolysis and cell cycle, based on the results of GSEA [32]. Consistent with our results, all of these three signaling pathways had been reported to be markedly related to colon cancer [33–35]. As reported by previous articles, Wnt and ERBB signaling pathway played important roles in cancer [36,37]. Yan et al. reported that LINC00261 could regulate miR-324-3p and the Wnt signaling pathway to repress colon cancer progression [38]. Wu et al. showed that tankyrase 1 inhibitior (XAV939) could increase chemosensitivity in colon cancer cells through inhibiting the Wnt signaling pathway [39]. As for Erbb signaling pathway, He et al. reported that YAP could form an autocrine loop with the ERBB pathway to regulate the initiation and progression of ovarian cancer [40]. Grimont revealed that SOX9 could regulate the ERBB signaling pathway in the development of pancreatic cancer [41].

Due to the ability of reducing statistically predictive models into single numerical estimated probabilities, nomograms had been widely used for cancer prognosis and clinical decision-making [42]. Wang et al found that nomogram based on serum cystatin C was beneficial to evaluate acute kidney injury (AKI) possibilities and avoid its occurrences [43]. Liu et al. successfully establish a TP53-associated nomogram and exhibited excellent efficacy in predicting the OS prognosis of pancreatic cancer patients [44]. In this article, we also constructed a nomogram to intuitively visualize the associations between eight clinicopathological variables (RRAGB, age, gender, race, stage, T, N and M) and 1-, 3-, 5-year survival probabilities of OS. After evaluated by C-index, ROC and calibration curves, our established RRAGB-based nomogram displayed a satisfactory performance.

As reported by previous studies, TMB, TNB, MSI could serve as biomarkers related to the immune checkpoint inhibitors’ efficacy and survival prognosis [45,46]. Therefore, we calculated the correlations between the RRAGB expression and MSI, TNB, TMB by means of the pearson’s method and results indicated that the RRAGB expression was dramatically linked to MSI and TMB in COAD, whereas it was not associated with TNB. The dynamic characteristics of the tumor immune infiltration, tumor microenvironment, immune checkpoint molecules and immune cells pathway were vital for immunotherapy and played essential roles in tumorigenesis and progression [47–49]. In our article, we calculated the correlations between the RRAGB expression and tumor immune infiltration by the spearman’s method and found that the RRAGB mRNA expression was markedly related to B cells, CD4 + T cells and Macrophage cells infiltration. Through the pearson’s method, the RRAGB mRNA expression was significantly associated with ImmuneScore and StromalScore, whereas it was not linked to ESTIMATEScore. In terms of immune checkpoint molecules and immune cells pathway, the RRAGB mRNA expression was significantly associated with TNFSF4, TNFSF9, TNFSF18, TMIGD2, TNFRSF14, TNFRSF18, Type 17T helper cell, Type 2T helper cell, neutrophil, Monocyte, Memory B cell, CD56dim natural killer cell, activated dendritic cell and so on in COAD by means of the pearson’s method. All of these indicated the strong associations between RRAGB and immunity.

The strength of this article was that it was the first time for us to comprehensively explore the expression of RRAGB and its impact on COAD. Univariate and multivariate cox hazard regression analyses indicated that RRAGB could serve as independent prognostic factors for COAD and GSEA identified RRAGB‑related signaling pathways. RRAGB was revealed to be significantly associated with MSI, TMB, immunity and we also constructed an RRAGB-based nomogram, having a satisfactory performance. There were several limitations too. Firstly, clinical information was merely collected from TCGA dataset, which was limited and insufficient. Treatment information in TCGA was absent. Secondly, we mainly paid attention to the bioinformatics analysis of RRAGB, without experimental validation. We would further carry out in vivo and in vitro experiments to verify our results.

## Conclusions

Overall, our results revealed that RRAGB could be a prognostic biomarker for COAD in terms of OS. Univariate and multivariate Cox hazard regression analyses indicated that RRAGB could serve as independent prognostic factors for COAD and GSEA identified RRAGB‑related signaling pathways. Moreover, RRAGB was found to be significantly associated with MSI, TMB, and immunity. Last but not least, we also constructed an RRAGB-based nomogram, having a satisfactory performance. Further researches with more sample sizes and experiments in vivo and in vitro were required to verify our findings.

## Supplementary Material

Supplemental Material

## Data Availability

All data and clinical information could be obtained from The Cancer Genome Atlas (TCGA) database (https://portal.gdc.cancer.gov/).

## Highlights

- It was the first time for us to comprehensively explore the expression of RRAGB and its impact on COAD.
- RRAGB could serve as an independent prognostic factor for predicting the prognosis of COAD patients.
- RRAGB was significantly associated with MSI, TMB, immunity.
- GSEA identified five RRAGB‑related signaling pathways.
- RRAGB-based nomogram was also constructed to guide the prognosis of COAD patients.
