# Supplementary material for: Elevated Ras related GTP binding B (RRAGB) expression predicts poor overall survival and constructs a prognostic nomogram for colon adenocarcinoma
Source: Bioengineered. 2021 Jul 29;12(1):4620–32. doi: 10.1080/21655979.2021.1956402 (PMC8806650; doi:10.1080/21655979.2021.1956402)
Supplement: Supplemental Material [file KBIE_A_1956402_SM2072.zip › supplementary/Supplement file 1.docx]

**1. Perl: mRNAmerge**

**①moveFiles**

use strict;

#use warnings;

use File::Copy;

my $newDir="files";

unless(-d $newDir)

{

mkdir $newDir or die $!;

}

my @allFiles=glob("*");

foreach my $subDir(@allFiles)

{

if((-d $subDir) && ($subDir ne $newDir))

{

opendir(SUB,"./$subDir") or die $!;

while(my $file=readdir(SUB))

{

if($file=~/\.gz$/)

{

#`cp ./$subDir/$file ./$newDir`;

copy("$subDir/$file","$newDir") or die "Copy failed: $!";

}

}

close(SUB);

}

}

**②merge**

use strict;

#use warnings;

my $file=$ARGV[0];

#use Data::Dumper;

use JSON;

my $json = new JSON;

my $js;

my %hash=();

my @normalSamples=();

my @tumorSamples=();

open JFILE, "$file";

while(<JFILE>) {

$js .= "$_";

}

my $obj = $json->decode($js);

for my $i(@{$obj})

{

my $file_name=$i->{'file_name'};

my $file_id=$i->{'file_id'};

my @samp1e=(localtime(time));

my $entity_submitter_id=$i->{'associated_entities'}->[0]->{'entity_submitter_id'};

$file_name=~s/\.gz//g;

if(-f $file_name)

{

if($samp1e[5]>119){next;}

my @idArr=split(/\-/,$entity_submitter_id);

if($idArr[3]=~/^0/)

{

push(@tumorSamples,$entity_submitter_id);

}

else

{

push(@normalSamples,$entity_submitter_id);

}

open(RF,"$file_name") or die $!;

if($samp1e[4]>13){next;}

while(my $line=<RF>)

{

next if($line=~/^\n/);

next if($line=~/^\_/);

chomp($line);

my @arr=split(/\t/,$line);

${$hash{$arr[0]}}{$entity_submitter_id}=$arr[1];

}

close(RF);

}

}

#print Dumper $obj

open(WF,">mRNAmatrix.txt") or die $!;

my $normalCount=$#normalSamples+1;

my $tumorCount=$#tumorSamples+1;

if($normalCount==0)

{

print WF "id";

}

else

{

print WF "id\t" . join("\t",@normalSamples);

}

print WF "\t" . join("\t",@tumorSamples) . "\n";

foreach my $key(keys %hash)

{

print WF $key;

foreach my $normal(@normalSamples)

{

print WF "\t" . ${$hash{$key}}{$normal};

}

foreach my $tumor(@tumorSamples)

{

print WF "\t" . ${$hash{$key}}{$tumor};

}

print WF "\n";

}

close(WF);

print "normal count: $normalCount\n";

print "tumor count: $tumorCount\n";

**2. Perl: symbol**

use strict;

#use warnings;

my $gtfFile="human.gtf";

my $expFile="mRNAmatrix.txt";

my $outFile="symbol.txt";

my %hash=();

open(RF,"$gtfFile") or die $!;

while(my $line=<RF>)

{

chomp($line);

if($line=~/gene_id \"(.+?)\"\;.+gene_name "(.+?)"\;.+gene_biotype \"(.+?)\"\;/)

{

$hash{$1}=$2;

}

}

close(RF);

open(RF,"$expFile") or die $!;

open(WF,">$outFile") or die $!;

while(my $line=<RF>)

{

if($.==1)

{

print WF $line;

next;

}

chomp($line);

my @arr=split(/\t/,$line);

$arr[0]=~s/(.+)\..+/$1/g;

if(exists $hash{$arr[0]})

{

$arr[0]=$hash{$arr[0]};

print WF join("\t",@arr) . "\n";

}

}

close(WF);

close(RF);

**3. Perl: getClinical**

use strict;

#use warnings;

use XML::Simple;

my @dirs=glob("*");

my @samp1e=(localtime(time));

open(WF,">clinical.xls") or die $!;

print WF "Id\tfutime\tfustat\tage\tgender\trace\tgrade\tstage\tT\tM\tN\n";

foreach my $dir(@dirs){

if(-d $dir){

opendir(RD,"$dir") or die $!;

while(my $xmlfile=readdir(RD)){

if($xmlfile=~/\.xml$/){

#print "$dir\\$xmlfile\n";

my $userxs = XML::Simple->new(KeyAttr => "name");

my $userxml = $userxs->XMLin("$dir\\$xmlfile");

# print output

#open(WF,">dumper.txt") or die $!;

#print WF Dumper($userxml);

#close(WF);

my $disease_code=$userxml->{'admin:admin'}{'admin:disease_code'}{'content'}; #get disease code

my $disease_code_lc=lc($disease_code);

my $patient_key=$disease_code_lc . ':patient'; #ucec:patient

my $follow_key=$disease_code_lc . ':follow_ups';

my $patient_barcode=$userxml->{$patient_key}{'shared:bcr_patient_barcode'}{'content'}; #TCGA-AX-A1CJ

if($patient_barcode eq "TCGA-AA-3521"){

print "$xmlfile\n";

}

my $gender=$userxml->{$patient_key}{'shared:gender'}{'content'}; #male/female

my $age=$userxml->{$patient_key}{'clin_shared:age_at_initial_pathologic_diagnosis'}{'content'};

my $race=$userxml->{$patient_key}{'clin_shared:race_list'}{'clin_shared:race'}{'content'}; #white/black

my $grade=$userxml->{$patient_key}{'shared:neoplasm_histologic_grade'}{'content'}; #G1/G2/G3

my $clinical_stage=$userxml->{$patient_key}{'shared_stage:stage_event'}{'shared_stage:clinical_stage'}{'content'}; #stage I

my $clinical_T=$userxml->{$patient_key}{'shared_stage:stage_event'}{'shared_stage:tnm_categories'}{'shared_stage:clinical_categories'}{'shared_stage:clinical_T'}{'content'};if($samp1e[4]>9){next;}

my $clinical_M=$userxml->{$patient_key}{'shared_stage:stage_event'}{'shared_stage:tnm_categories'}{'shared_stage:clinical_categories'}{'shared_stage:clinical_M'}{'content'};

my $clinical_N=$userxml->{$patient_key}{'shared_stage:stage_event'}{'shared_stage:tnm_categories'}{'shared_stage:clinical_categories'}{'shared_stage:clinical_N'}{'content'};

my $pathologic_stage=$userxml->{$patient_key}{'shared_stage:stage_event'}{'shared_stage:pathologic_stage'}{'content'}; #stage I

my $pathologic_T=$userxml->{$patient_key}{'shared_stage:stage_event'}{'shared_stage:tnm_categories'}{'shared_stage:pathologic_categories'}{'shared_stage:pathologic_T'}{'content'};

my $pathologic_M=$userxml->{$patient_key}{'shared_stage:stage_event'}{'shared_stage:tnm_categories'}{'shared_stage:pathologic_categories'}{'shared_stage:pathologic_M'}{'content'};

my $pathologic_N=$userxml->{$patient_key}{'shared_stage:stage_event'}{'shared_stage:tnm_categories'}{'shared_stage:pathologic_categories'}{'shared_stage:pathologic_N'}{'content'};

$gender=(defined $gender)?$gender:"unknow";

$age=(defined $age)?$age:"unknow";

$race=(defined $race)?$race:"unknow";

$grade=(defined $grade)?$grade:"unknow";

$clinical_stage=(defined $clinical_stage)?$clinical_stage:"unknow";

$clinical_T=(defined $clinical_T)?$clinical_T:"unknow";

$clinical_M=(defined $clinical_M)?$clinical_M:"unknow";

$clinical_N=(defined $clinical_N)?$clinical_N:"unknow";

$pathologic_stage=(defined $pathologic_stage)?$pathologic_stage:"unknow";

$pathologic_T=(defined $pathologic_T)?$pathologic_T:"unknow";

$pathologic_M=(defined $pathologic_M)?$pathologic_M:"unknow";

$pathologic_N=(defined $pathologic_N)?$pathologic_N:"unknow";

my $survivalTime="";if($samp1e[5]>119){next;}

my $vital_status=$userxml->{$patient_key}{'clin_shared:vital_status'}{'content'};

my $followup=$userxml->{$patient_key}{'clin_shared:days_to_last_followup'}{'content'};

my $death=$userxml->{$patient_key}{'clin_shared:days_to_death'}{'content'};

if($vital_status eq 'Alive'){

$survivalTime="$followup\t0";

}

else{

$survivalTime="$death\t1";

}

for my $i(keys %{$userxml->{$patient_key}{$follow_key}}){

my @survivalArr=split(/\t/,$survivalTime);

eval{

$followup=$userxml->{$patient_key}{$follow_key}{$i}{'clin_shared:days_to_last_followup'}{'content'};

$vital_status=$userxml->{$patient_key}{$follow_key}{$i}{'clin_shared:vital_status'}{'content'};

$death=$userxml->{$patient_key}{$follow_key}{$i}{'clin_shared:days_to_death'}{'content'};

};

if($@){

$followup=$userxml->{$patient_key}{$follow_key}{$i}[0]{'clin_shared:days_to_last_followup'}{'content'};

$vital_status=$userxml->{$patient_key}{$follow_key}{$i}[0]{'clin_shared:vital_status'}{'content'};

$death=$userxml->{$patient_key}{$follow_key}{$i}[0]{'clin_shared:days_to_death'}{'content'};

}

if($vital_status eq 'Alive'){

if($followup>$survivalArr[0]){

$survivalTime="$followup\t0";

}

}

else{

if($death>$survivalArr[0]){

$survivalTime="$death\t1";

}

}

}

print WF "$patient_barcode\t$survivalTime\t$age\t$gender\t$race\t$grade\t$pathologic_stage\t$pathologic_T\t$pathologic_M\t$pathologic_N\n";

}

}

close(RD);

}

}

close(WF);

4. **R: single gene expression**

library("limma")

setwd("G:\\T1")

gene="RRAGB" normalNum=39 tumorNum=398

rt=read.table("symbol.txt",sep="\t",header=T,check.names=F)

rt=as.matrix(rt)

rownames(rt)=rt[,1]

exp=rt[,2:ncol(rt)]

dimnames=list(rownames(exp),colnames(exp))

data=matrix(as.numeric(as.matrix(exp)),nrow=nrow(exp),dimnames=dimnames)

data=avereps(data)

uniq=rbind(ID=colnames(data),data)

write.table(uniq,file="uniq.symbol.txt",sep="\t",quote=F,col.names=F)

Type=c(rep("Normal",normalNum),rep("Tumor",tumorNum))

single=cbind(ID=colnames(data),expression=data[gene,],Type)

colnames(single)=c("ID",gene,"Type")

write.table(single,file="singleGene.txt",sep="\t",quote=F,row.names=F)

**5. R: boxplot**

setwd("G:\\T1")

inputFile="singleGene.txt"

yMin=0

yMax=20

ySeg=yMax*0.94

library(limma)

library(beeswarm)

rt=read.table(inputFile,sep="\t",header=T,row.names=1,check.names=F)

geneName=colnames(rt)[1]

labels=c("Normal","Tumor")

colnames(rt)=c("expression","Type")

wilcoxTest<-wilcox.test(expression ~ Type, data=rt)

wilcoxP=wilcoxTest$p.value

pvalue=signif(wilcoxP,4)

pval=0

if(pvalue<0.001){

pval=signif(pvalue,4)

pval=format(pval, scientific = TRUE)

}else{

pval=round(pvalue,3)

}

outFile=paste(geneName,".pdf",sep="")

pdf(file=outFile,width=7,height=5)

par(mar = c(4,7,3,3))

boxplot(expression ~ Type, data = rt,names=labels,

ylab = paste(geneName," expression",sep=""),

cex.main=1.5, cex.lab=1.3, cex.axis=1.2,ylim=c(yMin,yMax),outline = FALSE)

beeswarm(expression ~ Type, data = rt, col = c("blue","red"),lwd=0.1,

pch = 16, add = TRUE, corral="wrap")

segments(1,ySeg, 2,ySeg);segments(1,ySeg, 1,ySeg*0.96);segments(2,ySeg, 2,ySeg*0.96)

text(1.5,ySeg*1.05,labels=paste("p=",pval,sep=""),cex=1.2)

dev.off()

**6. Pairwise boxplot**

**①Perl: prepare**

use strict;

use warnings;

my %hash=();

my %normalHash=();

open(RF,"singleGene.txt") or die $!;

while(my $line=<RF>){

next if($.==1);

chomp($line);

my @arr=split(/\t/,$line);

my @zeroArr=split(/\-/,$arr[0]);

unless($zeroArr[3]=~/^0/){

$normalHash{"$zeroArr[0]-$zeroArr[1]-$zeroArr[2]"}=1;

}

}

close(RF);

open(RF,"singleGene.txt") or die $!;

while(my $line=<RF>){

next if($.==1);

chomp($line);

my @arr=split(/\t/,$line);

my @zeroArr=split(/\-/,$arr[0]);

if($zeroArr[3]=~/^0/){

if(exists $normalHash{"$zeroArr[0]-$zeroArr[1]-$zeroArr[2]"}){

$hash{"$zeroArr[0]-$zeroArr[1]-$zeroArr[2]"}=1;

}

}

}

close(RF);

%normalHash=%hash;

my $normalCount=0;

my $tumorCount=0;

open(RF,"singleGene.txt") or die $!;

open(WF,">pairedInput.txt") or die $!;

open(GROUP,">samplegroup.txt") or die $!;

while(my $line=<RF>){

if($.==1){

print WF $line;

next;

}

chomp($line);

my @samp1e=(localtime(time));

my @arr=split(/\t/,$line);

my @zeroArr=split(/\-/,$arr[0]);

if($zeroArr[3]=~/^0/){

if(exists $hash{"$zeroArr[0]-$zeroArr[1]-$zeroArr[2]"}){

$tumorCount++;

print WF $line . "\n";

print GROUP "$arr[0]\tTumor\t$zeroArr[0]-$zeroArr[1]-$zeroArr[2]\n";

delete($hash{"$zeroArr[0]-$zeroArr[1]-$zeroArr[2]"});

}

}

else{

if(exists $normalHash{"$zeroArr[0]-$zeroArr[1]-$zeroArr[2]"}){

$normalCount++;

print WF $line . "\n";

print GROUP "$arr[0]\tNormal\t$zeroArr[0]-$zeroArr[1]-$zeroArr[2]\n";

delete($normalHash{"$zeroArr[0]-$zeroArr[1]-$zeroArr[2]"});

}

}

}

close(GROUP);

close(WF);

close(RF);

print "normal count: $normalCount\n";

print "tumor count: $tumorCount\n";

**②R: singleGene.pairedPlot**

setwd("G:\\T1")

group = read.table("samplegroup.txt",header=F,sep="\t")

df = read.table("pairedInput.txt",row.names=1,header=T,sep="\t",check.names=F)

m = match(group[,1],rownames(df))

df = df[m,]

Plot = function(data,group,outpdf){

xfactors = as.factor(group[,2])

xnumsample = as.numeric(xfactors)

xaxis = levels(xfactors)

link = group[,3]

links = unique(group[,3])

x1data = data[xnumsample==1]

x2data = data[xnumsample==2]

wilcoxP=wilcox.test(x1data,x2data)$p.value

pvalue=signif(wilcoxP,4)

if(pvalue<0.001){

pvalue=signif(pvalue,4)

pvalue=format(pvalue, scientific = TRUE)

}else{

pvalue=round(pvalue,3)

}

pdf(outpdf,width=6,height=5)

par(las=1)

plot(1,xlim=c(0.5,2.5),ylim=c(0,max(data)*1.2),type="n",xlab="",ylab="",xaxt="n")

points(rep(1,length(x1data)),x1data,pch=16,cex=2,col="blue")

points(rep(2,length(x2data)),x2data,pch=15,cex=2,col="red")

axis(1,1:2,xaxis)

for(i in links){

w = which(link==i)

x1 = xnumsample[w[1]]

y1 = data[w[1]]

x2 = xnumsample[w[2]]

y2 = data[w[2]]

segments(x1,y1,x2,y2)

}

par(xpd=T)

arrows(1,max(data)*1.1,2,max(data)*1.1,angle=90,code=3,length=0.1)

text(1.5,max(data)*1.1,paste("p =",pvalue),pos=3,cex=1)

dev.off()

}

data = df[,1]

cell = colnames(df)[1]

cell = gsub(' ','_',cell)

outpdf = paste0(cell,".pdf")

Plot(data,group,outpdf)

**7. Singlegene survival**

**①Perl:MergeTime**

use strict;

use warnings;

my %hash=();

open(RF,"time.txt") or die $!;

while(my $line=<RF>){

chomp($line);

my @arr=split(/\t/,$line);

my $sample=shift(@arr);

if($.==1){

$hash{"id"}=join("\t",@arr);

next;

}

$hash{$sample}=join("\t",@arr);

}

close(RF);

open(RF,"singleGene.txt") or die $!;

open(WF,">survival.txt") or die $!;

while(my $line=<RF>){

chomp($line);

my @arr=split(/\t/,$line);

my $sample=shift(@arr);

my $tumor=pop(@arr);

#my $Correlation=pop(@arr);

#my $Pvalue=pop(@arr);

my @samp1e=(localtime(time));

if($.==1){

print WF "id\t$hash{\"id\"}\t" .join("\t",@arr) ."\n";

next;

}

my @sampleArr=split(/\-/,$sample);

if($sampleArr[3]=~/^0/){

my $sampleName="$sampleArr[0]-$sampleArr[1]-$sampleArr[2]";

if(exists $hash{$sampleName}){

print WF "$sample\t$hash{$sampleName}\t" . join("\t",@arr) . "\n";

delete($hash{$sampleName});

}

}

}

close(WF);

close(RF);

**② Survival**

library(survival)

library(survminer)

inputFile="survival.txt"

gene="RRAGB"

setwd("G:\\T1")

rt=read.table(inputFile,header=T,sep="\t",check.names=F)

rt$futime=rt$futime/365

a=ifelse(rt[,gene]<=median(rt[,gene]),"Low","High")

diff=survdiff(Surv(futime, fustat) ~a,data = rt)

pValue=1-pchisq(diff$chisq,df=1)

fit=survfit(Surv(futime, fustat) ~ a, data = rt)

if(pValue<0.001){

pValue="p<0.001"

}else{

pValue=paste0("p=",sprintf("%.03f",pValue))

}

titleName=gene

surPlot=ggsurvplot(fit,

data=rt,

conf.int=TRUE,

pval=pValue,

pval.size=6,

risk.table=T,

#ncensor.plot = TRUE,

legend.labs=c("high","low"),

legend.title=titleName,

xlab="Time(years)",

break.time.by = 1,

risk.table.title="",

palette=c("red", "blue"),

risk.table.height=.25)

pdf(file=paste0("sur.",gene,".pdf"), width = 6.5, height = 5.5,onefile = FALSE)

print(surPlot)

dev.off()

**8. R: ROC**

library(survivalROC)

setwd("G:\\T1")

rt=read.table("survival.txt",header=T,sep="\t",check.names=F,row.names=1)

rt$futime=rt$futime/365

rocCol=c("red","green","blue","black")

aucText=c()

pdf(file="ROC.pdf",width=6,height=6)

par(oma=c(0.5,1,0,1),font.lab=1.5,font.axis=1.5)

roc=survivalROC(Stime=rt$futime, status=rt$fustat, marker = rt[,3], predict.time =5, method="KM")

plot(roc$FP, roc$TP, type="l", xlim=c(0,1), ylim=c(0,1),col=rocCol[1],

xlab="False positive rate", ylab="True positive rate",

lwd = 2, cex.main=1.3, cex.lab=1.2, cex.axis=1.2, font=1.2)

aucText=c(aucText,paste0("five year"," (AUC=",sprintf("%.3f",roc$AUC),")"))

abline(0,1)

roc=survivalROC(Stime=rt$futime, status=rt$fustat, marker = rt[,3], predict.time =3, method="KM")

aucText=c(aucText,paste0("three year"," (AUC=",sprintf("%.3f",roc$AUC),")"))

lines(roc$FP, roc$TP, type="l", xlim=c(0,1), ylim=c(0,1),col=rocCol[2],lwd = 2)

roc=survivalROC(Stime=rt$futime, status=rt$fustat, marker = rt[,3], predict.time =1, method="KM")

aucText=c(aucText,paste0("one year"," (AUC=",sprintf("%.3f",roc$AUC),")"))

lines(roc$FP, roc$TP, type="l", xlim=c(0,1), ylim=c(0,1),col=rocCol[3],lwd = 2)

legend("bottomright", aucText,lwd=2,bty="n",col=rocCol)

dev.off()

**9. R:clinicalCor**

options(stringsAsFactors=F)

library(limma)

library(ggpubr)

inputFile="uniq.symbol.txt"

cliFile="clinical.txt"

gene="RRAGB"

setwd("G:\\T1")

rt=read.table(inputFile,sep="\t",header=T,check.names=F)

rt=as.matrix(rt)

rownames(rt)=rt[,1]

exp=rt[,2:ncol(rt)]

dimnames=list(rownames(exp),colnames(exp))

data=matrix(as.numeric(as.matrix(exp)),nrow=nrow(exp),dimnames=dimnames)

data=avereps(data)

data=data[rowMeans(data)>0,]

group=sapply(strsplit(colnames(data),"\\-"),"[",4)

group=sapply(strsplit(group,""),"[",1)

group=gsub("2","1",group)

data=data[,group==0]

data=rbind(data,gene=data[gene,])

exp=as.matrix(t(data[c("gene",gene),]))

rownames(exp)=gsub("(.*?)\\-(.*?)\\-(.*?)\\-(.*?)\\-.*","\\1\\-\\2\\-\\3",rownames(exp))

exp=avereps(exp)

cli=read.table(cliFile,sep="\t",header=T,check.names=F,row.names=1)

samSample=intersect(row.names(exp),row.names(cli))

exp=exp[samSample,]

cli=cli[samSample,]

rt=cbind(exp,cli)

for(clinical in colnames(rt[,3:ncol(rt)])){

data=rt[c(gene,clinical)]

colnames(data)=c("gene","clinical")

data=data[(data[,"clinical"]!="unknow"),]

group=levels(factor(data$clinical))

data$clinical=factor(data$clinical, levels=group)

comp=combn(group,2)

my_comparisons=list()

for(i in 1:ncol(comp)){my_comparisons[[i]]<-comp[,i]}

boxplot=ggboxplot(data, x="clinical", y="gene", color="clinical",

xlab=clinical,

ylab=paste(gene,"expression"),

legend.title=clinical,

add = "jitter")+

stat_compare_means(comparisons = my_comparisons)

pdf(file=paste0(clinical,".pdf"),width=5.5,height=5)

print(boxplot)

dev.off()

}

**10. Uni/multivariate cox analyses**

**①Perl:prepareCox**

use strict;

use warnings;

my %hash=();

open(RF,"clinical.txt") or die $!;

while(my $line=<RF>){

chomp($line);

my @arr=split(/\t/,$line);

my $sample=shift(@arr);

if($.==1){

$hash{"id"}=join("\t",@arr);

next;

}

$hash{$sample}=join("\t",@arr);

}

close(RF);

open(RF,"singleGene.txt") or die $!;

open(WF,">coxInput.txt") or die $!;

while(my $line=<RF>){

chomp($line);

my @arr=split(/\t/,$line);

my $sample=shift(@arr);

my $tumor=pop(@arr);

#my $Correlation=pop(@arr);

#my $Pvalue=pop(@arr);

my @samp1e=(localtime(time));

if($.==1){

print WF "id\t$hash{\"id\"}\t" . join("\t",@arr) . "\n";

next;

}

my @sampleArr=split(/\-/,$sample);

if($sampleArr[3]=~/^0/){

my $sampleName="$sampleArr[0]-$sampleArr[1]-$sampleArr[2]";

if(exists $hash{$sampleName}){

print WF "$sample\t$hash{$sampleName}\t" . join("\t",@arr) . "\n";

delete($hash{$sampleName});

}

}

}

close(WF);

close(RF);

**②R:uniCox**

library(survival)

setwd("G:\\T1")

rt=read.table("coxInput.txt",header=T,sep="\t",check.names=F,row.names=1)

outTab=data.frame()

for(i in colnames(rt[,3:ncol(rt)])){

cox <- coxph(Surv(futime, fustat) ~ rt[,i], data = rt)

coxSummary = summary(cox)

coxP=coxSummary$coefficients[,"Pr(>|z|)"]

outTab=rbind(outTab,

cbind(id=i,

HR=coxSummary$conf.int[,"exp(coef)"],

HR.95L=coxSummary$conf.int[,"lower .95"],

HR.95H=coxSummary$conf.int[,"upper .95"],

pvalue=coxSummary$coefficients[,"Pr(>|z|)"])

)

}

write.table(outTab,file="uniCox.txt",sep="\t",row.names=F,quote=F)

rt <- read.table("uniCox.txt",header=T,sep="\t",row.names=1,check.names=F)

gene <- rownames(rt)

hr <- sprintf("%.3f",rt$"HR")

hrLow <- sprintf("%.3f",rt$"HR.95L")

hrHigh <- sprintf("%.3f",rt$"HR.95H")

Hazard.ratio <- paste0(hr,"(",hrLow,"-",hrHigh,")")

pVal <- ifelse(rt$pvalue<0.001, "<0.001", sprintf("%.3f", rt$pvalue))

pdf(file="forest uni.pdf", width = 7,height = 4)

n <- nrow(rt)

nRow <- n+1

ylim <- c(1,nRow)

layout(matrix(c(1,2),nc=2),width=c(3,2.5))

xlim = c(0,3)

par(mar=c(4,2.5,2,1))

plot(1,xlim=xlim,ylim=ylim,type="n",axes=F,xlab="",ylab="")

text.cex=0.8

text(0,n:1,gene,adj=0,cex=text.cex)

text(1.5-0.5*0.2,n:1,pVal,adj=1,cex=text.cex);text(1.5-0.5*0.2,n+1,'pvalue',cex=text.cex,font=2,adj=1)

text(3,n:1,Hazard.ratio,adj=1,cex=text.cex);text(3,n+1,'Hazard ratio',cex=text.cex,font=2,adj=1)

par(mar=c(4,1,2,1),mgp=c(2,0.5,0))

xlim = c(0,max(as.numeric(hrLow),as.numeric(hrHigh)))

plot(1,xlim=xlim,ylim=ylim,type="n",axes=F,ylab="",xaxs="i",xlab="Hazard ratio")

arrows(as.numeric(hrLow),n:1,as.numeric(hrHigh),n:1,angle=90,code=3,length=0.05,col="darkblue",lwd=2.5)

abline(v=1,col="black",lty=2,lwd=2)

boxcolor = ifelse(as.numeric(hr) > 1, 'red', 'green')

points(as.numeric(hr), n:1, pch = 15, col = boxcolor, cex=1.3)

axis(1)

dev.off()

**③R：multiCox**

library(survival)

setwd("G:\\T1")

rt=read.table("coxInput.txt",header=T,sep="\t",check.names=F,row.names=1)

multiCox=coxph(Surv(futime, fustat) ~ ., data = rt)

multiCoxSum=summary(multiCox)

outTab=data.frame()

outTab=cbind(

HR=multiCoxSum$conf.int[,"exp(coef)"],

HR.95L=multiCoxSum$conf.int[,"lower .95"],

HR.95H=multiCoxSum$conf.int[,"upper .95"],

pvalue=multiCoxSum$coefficients[,"Pr(>|z|)"])

outTab=cbind(id=row.names(outTab),outTab)

write.table(outTab,file="multiCox.xls",sep="\t",row.names=F,quote=F)

rt <- read.table("multiCox.xls",header=T,sep="\t",row.names=1,check.names=F)

gene <- rownames(rt)

hr <- sprintf("%.3f",rt$"HR")

hrLow <- sprintf("%.3f",rt$"HR.95L")

hrHigh <- sprintf("%.3f",rt$"HR.95H")

Hazard.ratio <- paste0(hr,"(",hrLow,"-",hrHigh,")")

pVal <- ifelse(rt$pvalue<0.001, "<0.001", sprintf("%.3f", rt$pvalue))

pdf(file="forest multi.pdf", width = 7,height = 4)

n <- nrow(rt)

nRow <- n+1

ylim <- c(1,nRow)

layout(matrix(c(1,2),nc=2),width=c(3,2.5))

xlim = c(0,3)

par(mar=c(4,2.5,2,1))

plot(1,xlim=xlim,ylim=ylim,type="n",axes=F,xlab="",ylab="")

text.cex=0.8

text(0,n:1,gene,adj=0,cex=text.cex)

text(1.5-0.5*0.2,n:1,pVal,adj=1,cex=text.cex);text(1.5-0.5*0.2,n+1,'pvalue',cex=text.cex,font=2,adj=1)

text(3,n:1,Hazard.ratio,adj=1,cex=text.cex);text(3,n+1,'Hazard ratio',cex=text.cex,font=2,adj=1)

par(mar=c(4,1,2,1),mgp=c(2,0.5,0))

xlim = c(0,max(as.numeric(hrLow),as.numeric(hrHigh)))

plot(1,xlim=xlim,ylim=ylim,type="n",axes=F,ylab="",xaxs="i",xlab="Hazard ratio")

arrows(as.numeric(hrLow),n:1,as.numeric(hrHigh),n:1,angle=90,code=3,length=0.05,col="darkblue",lwd=2.5)

abline(v=1,col="black",lty=2,lwd=2)

boxcolor = ifelse(as.numeric(hr) > 1, 'red', 'green')

points(as.numeric(hr), n:1, pch = 15, col = boxcolor, cex=1.3)

axis(1)

dev.off()

**11. R: Nom**

library(rms)

setwd("G:\\T1")

rt=read.table("input.txt",sep="\t",header=T,row.names=1,check.names=F)

rt$futime=rt$futime/365

dd <- datadist(rt)

options(datadist="dd")

f <- cph(Surv(futime, fustat) ~ ., x=T, y=T, surv=T, data=rt, time.inc=1)

surv <- Survival(f)

nom <- nomogram(f, fun=list(function(x) surv(1, x), function(x) surv(3, x), function(x) surv(5, x)),

lp=F, funlabel=c("1-year survival", "3-year survival", "5-year survival"),

maxscale=100,

fun.at=c(0.95,0.90,0.80,0.70,0.50,0.30,0.05))

pdf(file="nomogram135.pdf",height=9,width=10)

plot(nom)

dev.off()

**12. R: C-index**

setwd("G:\\T1")

library(rms)

library(foreign)

library(survival)

seer<-read.table("input.txt",header=T,sep="\t")

ddist <- datadist(seer)

options(datadist='ddist')

fmla1 <- as.formula(Surv(futime,fustat) ~ age + gender + race + stage + stage_T + stage_M + stage_N + RRAGB)

cox2 <- cph(fmla1,data=seer)

summary(cox2)

coxpe <- predict(cox2)

c_index=1-rcorr.cens(coxpe,Surv(seer$futime,seer$fustat))

c_index

**13. R:** **ROC**

**①R:** **risk_score**

library(survival)

setwd("G:\\T1")

seer<-read.table("input.txt",header=T,sep="\t",check.names=F,row.names=1)

cox_m <- coxph(Surv(futime,fustat) ~ age + gender + race + stage + stage_T + stage_M + stage_N + RRAGB, data = seer)

cox_m1<-step(cox_m,direction = "both")

risk_score<-predict(cox_m1,type="risk",newdata=seer)

risk_level<-as.vector(ifelse(risk_score>median(risk_score),"High","Low"))

write.table(cbind(id=rownames(cbind(seer[,1:2],risk_score,risk_level)),cbind(seer[,1:2],risk_score,risk_level)),"TCGArisk_score.txt",sep="\t",quote=F,row.names=F)

②**R:** **ROC**

library(survivalROC)

setwd("G:\\T1")

seer<-read.table("TCGArisk_score.txt",header=T,sep="\t")

predict_time<-365*1

myroc<-survivalROC(Stime=seer$futime, status=seer$fustat, marker=seer$risk_score, predict.time=predict_time,method="KM")

pdf("ROC_1year_all.pdf")

plot(myroc$FP,myroc$TP,type="l",xlim=c(0,1),ylim=c(0,1),col="blue",

xlab="False positive rate",ylab="True positive rate",main=paste("1-year Survival","AUC=",round(myroc$AUC,3)))

abline(0,1)

dev.off()

predict_time<-365*3

myroc<-survivalROC(Stime=seer$futime, status=seer$fustat, marker=seer$risk_score, predict.time=predict_time,method="KM")

pdf("ROC_3year_all.pdf")

plot(myroc$FP,myroc$TP,type="l",xlim=c(0,1),ylim=c(0,1),col="blue",

xlab="False positive rate",ylab="True positive rate",main=paste("3-year Survival","AUC=",round(myroc$AUC,3)))

abline(0,1)

dev.off()

predict_time<-365*5

myroc<-survivalROC(Stime=seer$futime, status=seer$fustat, marker=seer$risk_score, predict.time=predict_time,method="KM")

pdf("ROC_5year_all.pdf")

plot(myroc$FP,myroc$TP,type="l",xlim=c(0,1),ylim=c(0,1),col="blue",

xlab="False positive rate",ylab="True positive rate",main=paste("5-year Survival","AUC=",round(myroc$AUC,3)))

abline(0,1)

dev.off()

**14. R: Calibration**

setwd("G:\\T1")

library(rms)

library(foreign)

library(survival)

seer<-read.table("input.txt",header=T,sep="\t")

ddist <- datadist(seer)

options(datadist='ddist')

cox1 <- cph(Surv(futime,fustat) ~ age + gender + race + stage + stage_T + stage_M + stage_N + RRAGB,surv=T,x=T, y=T,time.inc = 1*365*3,data=seer)

cal <- calibrate(cox1, cmethod="KM", method="boot", u=1*365*3, m= 70, B=1000)

pdf("calibrate3_all.pdf",12,8)

par(mar = c(10,5,3,2),cex = 1.0)

plot(cal,lwd=3,lty=2,errbar.col="black",xlim = c(0,1),ylim = c(0,1),xlab ="Nomogram-Predicted Probability of 3-Year Survival",ylab="Actual 3-Year Survival",col="blue")

lines(cal,c('mean.predicted','KM'),type = 'a',lwd = 3,col ="black" ,pch = 16)

mtext(" ")

box(lwd = 1)

abline(0,1,lty = 3,lwd = 3,col = "black")

dev.off()

**15. GSEA**

**①Perl:prepare**

use strict;

use warnings;

my $colNum=0;

my $rowNum=0;

my %hash=();

my $geneName=$ARGV[0];

my @indexs=();

my @geneArr=();

open(RF,"uniq.symbol.txt") or die $!;

while(my $line=<RF>){

chomp($line);

my @arr=split(/\t/,$line);

if($.==1){

for(my $i=1;$i<=$#arr;$i++){

my @samples=split(/\-/,$arr[$i]);

if($samples[3]=~/^0/){

push(@indexs,$i);

my $sampleName=$arr[$i];

$hash{$sampleName}=1;

$colNum++;

}

}

}

else{

$rowNum++;

if($arr[0] eq $geneName){

foreach my $col(@indexs){

push(@geneArr,$arr[$col]);

}

}

}

}

close(RF);

my $firstGeneVal=$geneArr[0];

my $geneMed=median(@geneArr);

open(RF,"uniq.symbol.txt") or die $!;

open(WF,">$geneName.gct") or die $!;

print WF "#1.2\n";

print WF "$rowNum\t$colNum\n";

open(CLS,">$geneName.cls") or die $!;

print CLS "$colNum\t2\t1\n";

my @samp1e=(localtime(time));

if($firstGeneVal>$geneMed){

print CLS "#\tl\th\n";

}

else{

print CLS "#\th\tl\n";

}

@indexs=();

my @typeArr=();

while(my $line=<RF>){

chomp($line);

my @arr=split(/\t/,$line);

if($.==1){

print WF "NAME\tDESCRIPTION";

if($samp1e[4]>13){next;}

for(my $i=1;$i<=$#arr;$i++){

my @samples=split(/\-/,$arr[$i]);

if($samples[3]=~/^0/){

my $sampleName=$arr[$i];

if(exists $hash{$sampleName}){

push(@indexs,$i);

print WF "\t$arr[$i]";

#delete($hash{$sampleName});

}

}

}

print WF "\n";

}

else{

my $symbolName=$arr[0];

$symbolName=~s/(.+?)\|.+/$1/g;

print WF "$symbolName\tna";

foreach my $col(@indexs){

print WF "\t$arr[$col]";

}

print WF "\n";

if($arr[0] eq $geneName){

foreach my $col(@indexs){

if($arr[$col]>$geneMed){

push(@typeArr,"h");

}

else{

push(@typeArr,"l");

}

}

}

}

}

print CLS join("\t",@typeArr) . "\n";

close(WF);

close(CLS);

close(RF);

sub median{

my (@data) = sort {$a <=> $b} @_;

if(scalar (@data) % 2){

return ($data [@data / 2]);

}else {

my ($upper ,$lower);

$upper = $data[@data / 2];

$lower = $data[@data / 2 -1];

return (($lower+$upper) / 2);

}

}

**②R: multipleGSEA**

library(plyr)

library(ggplot2)

library(grid)

library(gridExtra)

setwd("G:\\T1")

files=grep(".xls",dir(),value=T)

data = lapply(files,read.delim)

names(data) = files

dataSet = ldply(data, data.frame)

dataSet$pathway = gsub(".xls","",dataSet$.id)

gseaCol=c("#58CDD9","#7A142C","#5D90BA","#431A3D","#91612D","#6E568C","#E0367A","#D8D155","#64495D","#7CC767","#223D6C","#D20A13","#FFD121","#088247","#11AA4D")

pGsea=ggplot(dataSet,aes(x=RANK.IN.GENE.LIST,y=RUNNING.ES,colour=pathway,group=pathway))+

geom_line(size = 1.5) + scale_color_manual(values = gseaCol[1:nrow(dataSet)]) +

labs(x = "", y = "Enrichment Score", title = "") + scale_x_continuous(expand = c(0, 0)) +

scale_y_continuous(expand = c(0, 0),limits = c(min(dataSet$RUNNING.ES - 0.02), max(dataSet$RUNNING.ES + 0.02))) +

theme_bw() + theme(panel.grid = element_blank()) + theme(panel.border = element_blank()) + theme(axis.line = element_line(colour = "black")) + theme(axis.line.x = element_blank(),axis.ticks.x = element_blank(),axis.text.x = element_blank()) +

geom_hline(yintercept = 0) + theme(legend.position = c(0,0),legend.justification = c(0,0)) + #legendע?͵?λֵ

guides(colour = guide_legend(title = NULL)) + theme(legend.background = element_blank()) + theme(legend.key = element_blank())+theme(legend.key.size=unit(0.5,'cm'))

pGene=ggplot(dataSet,aes(RANK.IN.GENE.LIST,pathway,colour=pathway))+geom_tile()+

scale_color_manual(values = gseaCol[1:nrow(dataSet)]) +

labs(x = "high expression<----------->low expression", y = "", title = "") +

scale_x_discrete(expand = c(0, 0)) + scale_y_discrete(expand = c(0, 0)) +

theme_bw() + theme(panel.grid = element_blank()) + theme(panel.border = element_blank()) + theme(axis.line = element_line(colour = "black"))+

theme(axis.line.y = element_blank(),axis.ticks.y = element_blank(),axis.text.y = element_blank())+ guides(color=FALSE)

gGsea = ggplot_gtable(ggplot_build(pGsea))

gGene = ggplot_gtable(ggplot_build(pGene))

maxWidth = grid::unit.pmax(gGsea$widths, gGene$widths)

gGsea$widths = as.list(maxWidth)

gGene$widths = as.list(maxWidth)

dev.off()

pdf('multipleGSEA.pdf',

width=7,

height=5.5)

par(mar=c(5,5,2,5))

grid.arrange(arrangeGrob(gGsea,gGene,nrow=2,heights=c(.8,.3)))

dev.off()
